# Clinical Characterization of Host Response to Simian Hemorrhagic Fever Virus Infection in Permissive and Refractory Hosts: A Model for Determining Mechanisms of VHF Pathogenesis

**DOI:** 10.3390/v11010067

**Published:** 2019-01-15

**Authors:** Joseph P. Cornish, Ian N. Moore, Donna L. Perry, Abigail Lara, Mahnaz Minai, Dominique Promeneur, Katie R. Hagen, Kimmo Virtaneva, Monica Paneru, Connor R. Buechler, David H. O’Connor, Adam L. Bailey, Kurt Cooper, Steven Mazur, John G. Bernbaum, James Pettitt, Peter B. Jahrling, Jens H. Kuhn, Reed F. Johnson

**Affiliations:** 1 Emerging Viral Pathogens Section, Laboratory of Immunoregulation, Division of Intramural Research, National Institute of Allergy and Infectious Diseases, National Institutes of Health, 8200 Research Plaza, Fort Detrick, Frederick, MD 21702, USA; joseph.cornish@nih.gov (J.P.C.); jahrlingp@niaid.nih.gov (P.B.J.); 2 Infectious Disease Pathogenesis Section, Comparative Medicine Branch, Division of Intramural Research, National Institute of Allergy and Infectious Diseases, National Institutes of Health, 12735 Twinbrook Parkway, Rockville, MD 20852, USA; Ian.Moore@NIH.gov (I.N.M.); minaim@niaid.nih.gov (M.M.); 3 Integrated Research Facility, Division of Clinical Research, National Institute of Allergy and Infectious Diseases, National Institutes of Health, 8200 Research Plaza, Fort Detrick, Frederick, MD 21702, USA; donna.perry@NIH.gov (D.L.P.); laraa@MedImmune.com (A.L.); dominique.promeneur@nih.gov (D.P.); katie.hagen@nih.gov (K.R.H.); kurt.cooper@nih.gov (K.C.); steven.mazur@nih.gov (S.M.); bernbaumjg@niaid.nih.gov (J.G.B.); james.d.pettitt@gmail.com (J.P.); kuhnjens@niaid.nih.gov (J.H.K.); 4 Genomics Unit, Research Technologies Branch, Rocky Mountain Laboratories, National Institute of Allergy and Infectious Diseases, National Institutes of Health, 903 South 4th Street, Hamilton, MT 59840, USA; kvirtaneva@niaid.nih.gov (K.V.); monica.paneru@nih.gov (M.P.); 5 Department of Pathology and Laboratory Medicine, University of Wisconsin-Madison, 3170 UW Medical Foundation Centennial Building, 1685 Highland Ave, Madison, WI 53711, USA; connor.buechler@gmail.com (C.R.B.); dhoconno@wisc.edu (D.H.O.); adam.bailey@wustl.edu (A.L.B.); 6 Wisconsin National Primate Research Center, Southwest Commuter Path, Madison, WI 53711, USA

**Keywords:** *Arteriviridae*, arterivirus, host response, macaque, patas monkey, pathogenesis, SHFV, simarterivirus, simian hemorrhagic fever, viral hemorrhagic fever

## Abstract

Simian hemorrhagic fever virus (SHFV) causes a fulminant and typically lethal viral hemorrhagic fever (VHF) in macaques (Cercopithecinae: *Macaca* spp.) but causes subclinical infections in patas monkeys (Cercopithecinae: *Erythrocebus patas*). This difference in disease course offers a unique opportunity to compare host responses to infection by a VHF-causing virus in biologically similar susceptible and refractory animals. Patas and rhesus monkeys were inoculated side-by-side with SHFV. Unlike the severe disease observed in rhesus monkeys, patas monkeys developed a limited clinical disease characterized by changes in complete blood counts, serum chemistries, and development of lymphadenopathy. Viral RNA was measurable in circulating blood 2 days after exposure, and its duration varied by species. Infectious virus was detected in terminal tissues of both patas and rhesus monkeys. Varying degrees of overlap in changes in serum concentrations of interferon (IFN)-γ, monocyte chemoattractant protein (MCP)-1, and interleukin (IL)-6 were observed between patas and rhesus monkeys, suggesting the presence of common and species-specific cytokine responses to infection. Similarly, quantitative immunohistochemistry of livers from terminal monkeys and whole blood flow cytometry revealed varying degrees of overlap in changes in macrophages, natural killer cells, and T-cells. The unexpected degree of overlap in host response suggests that relatively small subsets of a host’s response to infection may be responsible for driving hemorrhagic fever pathogenesis. Furthermore, comparative SHFV infection in patas and rhesus monkeys offers an experimental model to characterize host–response mechanisms associated with viral hemorrhagic fever and evaluate pan-viral hemorrhagic fever countermeasures.

## 1. Introduction

Viral hemorrhagic fevers (VHFs) are primarily caused by single-stranded RNA viruses [1]. VHF is a broadly defined syndrome: fever, hepatic and renal complications, large increases in proinflammatory cytokines, and coagulopathy are common features [2,3]. Simian hemorrhagic fever virus (SHFV) is a positive-sense, single-stranded RNA virus classified in the family *Arteriviridae*, which also includes equine arteritis virus and porcine reproductive and respiratory syndrome viruses 1 and 2 [4,5]. In addition to SHFV, several other simian arteriviruses (subfamily *Simarterivirinae*) have been identified [6,7,8]. Among simarteriviruses, SHFV, simian hemorrhagic encephalitis virus (SHEV), and Pebjah virus (PBJV) are known to cause severe disease in Asian macaques of various species [9]. Kibale red colobus virus 1 (KRCV-1) was found to cause a self-limiting disease in crab-eating macaques (*Macaca fascicularis*) [10]. It is not known whether the other identified simarteriviruses infect or cause disease in macaques or their natural hosts. Here, and in combination with an article by Buechler et al. [11], we examine infection of natural hosts (patas monkeys [*Erythrocebus patas*] and olive baboons [*Papio anubis*]) of simarteriviruses and compare disease course of these simarteriviruses in rhesus monkeys (*Macaca mulatta*).

SHFV was discovered during a VHF outbreak of simian hemorrhagic fever (SHF) in National Institutes of Health (NIH) animal facilities in the United States in 1964 [12]. Transmission during the NIH SHF outbreak is thought to have occurred through tattooing needles used on both macaques and co-housed African primates [12]. The virus is highly virulent in rhesus monkeys, crab-eating macaques, stump-tailed macaques (*Macaca arctoides*), and Japanese macaques (*Macaca fuscata*), but SHFV is suspected to cause little to no disease in African primates such as patas monkeys or baboons [11,12,13,14]. SHFV infection in macaques mirrors aspects of human VHFs, such as Ebola virus disease, by inducing fever, edema, coagulopathy, hepatocellular degeneration and necrosis, and elevated inflammatory cytokine concentrations [13,14,15]. Like all VHFs, SHF is thought to be driven by a dysregulated host response leading to a dysregulated immune response and poor viral clearance [13,14,16].

Studying SHF offers a unique opportunity to compare infection and associated responses in refractory and highly susceptible primates that are biologically similar to each other and to humans. Some hemorrhagic fever-causing viruses naturally infect non-primate mammals that may serve as hosts or reservoirs. For instance, Marburg virus and Ravn virus (*Filoviridae: Marburgvirus*) naturally circulate in Egyptian rousettes (*Rousettus aegyptiacus*), in which they do not cause disease, whereas these viruses cause frequently lethal disease experimentally in primates and naturally in humans [17]. Similarly, arenaviruses associated with human VHFs, such as Machupo virus and Lassa virus (*Arenaviridae: Mammarenavirus*), subclinically infect distinct rodent reservoir hosts [18,19]. Comparing the response to infection between refractory hosts, preferably the reservoirs themselves, and susceptible hosts may offer significant insight into responses involved in VHF pathogenesis. For most hemorrhagic fever-causing viruses, comparisons between refractory and susceptible animals during infection is confounded by large biological differences. For example, work with pathogenic mammarenaviruses has demonstrated that mechanisms present in murine hosts, even susceptible ones, are fundamentally different than those of primates [20]. Unlike these examples, SHFV infects biologically similar refractory and susceptible animals and may provide a path towards meaningful interspecies comparisons of responses to hemorrhagic fever-causing virus infection.

The goal of this work was to compare host responses in biologically similar nonhuman primates, patas (refractory) monkeys and rhesus (susceptible) monkeys, infected with SHFV to identify factors associated with differential outcomes to infection. The findings are the first in-depth characterization of SHFV infection in patas monkeys and confirm that patas monkeys are largely unaffected by SHFV infection. Our work demonstrates that, although patas and rhesus monkeys develop drastically different diseases, the host responses to infection overlap, and suggest that VHF pathogenesis may be initiated by relatively small perturbations to the host’s response to infection.

## 2. Materials and Methods

### 2.1. Cells and Virus

Simian hemorrhagic fever virus (SHFV; *Nidovirales*: *Arteriviridae*: *Simarterivirinae*: *Deltaarterivirus*) strain LVR42-0/M6941 [21] was passaged twice before a final passage on MA-104 cells to create the virus stock. Briefly, the virus stock was prepared by freeze-thawing infected cells three times prior to clarification with low-speed centrifugation and was then concentrated by centrifugation at 16,000× *g*. The pellets were resuspended in PBS and combined. The final viral stock was sequenced as in [22] for quality control purposes (GenBank #MH155201). Sequencing confirmed the expected genotype and lack of any contamination.

### 2.2. Animals

Six patas monkeys (4 females and 2 males) and 6 rhesus monkeys (3 females and 3 males) were used in this study. The patas monkeys ranged from 5.51–14.01 kg in weight and 9–14 years in age, whereas rhesus monkeys ranged from 4.77–12.75 kg in weight and 8–12 years in age. Rhesus monkeys were obtained from the National Institute of Allergy and Infectious Disease (NIH/NIAID) rhesus monkey colony. The patas monkeys were obtained from the National Institute of Child Health and Human Development (NIH/NICHD). The subjects were screened for simian T-lymphotrophic virus, simian immunodeficiency virus, and simian retrovirus infections and cleared for use in the experiment by the facility veterinarian. The patas monkeys were determined to be serologically negative for SHFV prior to enrollment. The rhesus monkeys were obtained from a SHFV-free source, and, therefore, were not screened prior to use in this experiment. The subjects were randomly assigned to 4 groups (2 groups of patas monkeys, inoculated and mock-inoculated, and 2 groups of rhesus monkeys, inoculated and mock-inoculated), for sex, age and weight. The animals of 1 group of patas monkeys and 1 group of rhesus monkeys each received 5000 PFU of SHFV diluted in 1 mL of phosphate-buffered saline (PBS), whereas the animals of the remaining groups each received 1 mL of PBS (mock) by intramuscular injection of the right quadriceps. The subjects were housed in separate rooms and had access to food and water ad libitum.

The subjects were monitored at least twice daily. Physical exams were performed on pre-determined experimental days (−9, −6, 0, 2, 4, 6, 8, 10, 12, 15, 19, and 21) and prior to euthanasia. Blood was collected on all the physical exam days, except day 0, and prior to euthanasia. The scheduled days for euthanasia with necropsy were as follows: mock-inoculated patas monkeys at 19 days post-inoculation (PI), SHFV-inoculated patas monkeys at 21 days post-inoculation and SHFV-inoculated rhesus monkeys at 20 days post-inoculation. Mock-inoculated rhesus monkeys were euthanized and underwent complete necropsies at 10 days post-inoculation as SHFV-inoculated rhesus monkeys typically succumb on or around day 10 PI [13]. The staggering of planned euthanasia days was logistically necessary to ensure safe working conditions in biosafety level 4 (BSL-4) working environments. The subjects were euthanized at scheduled times or upon reaching pre-established clinical endpoint criteria (score). The clinical score was assessed across 5 categories (overall clinical appearance, respiratory function, responsiveness, and core body temperature) with a score of 0 (normal) to 10 (most severe disease). If the total score for all 5 categories, or the score of any 1 category, was 10 or greater, then the animal was euthanized. At euthanasia, the subjects were perfused with saline before necropsy and sample collection. The subjects were housed in an Association for Assessment and Accreditation of Laboratory Animal Care (AAALAC) International-accredited facility under BSL-4 conditions. All the experimental procedures were approved by the NIAID Division of Clinical Research (DCR), Animal Care and Use Committee and were performed in compliance with the Animal Welfare Act regulations, Public Health Service policy, and the Guide for the Care and Use of Laboratory Animals recommendations.

### 2.3. Virus Quantification

Virus stock and tissue concentrations were determined by plaque assay on grivet (*Chlorocebus aethiops*) kidney epithelial MA-104 cells (ATCC, Manassas, VA, USA). Briefly, serial dilutions of 10% (*w*/*v*) tissue homogenates were added to the cell monolayers and incubated for 1 h. Then, the monolayers were overlaid with 0.8% tragacanth (Sigma, St. Louis, MO, USA), minimal essential medium (Lonza, Walkersville, MD, USA), 1% penicillin–streptomycin (Lonza, Basel, Switzerland), and 2% heat-inactivated fetal bovine serum (Sigma, St. Louis, MO, USA) final concentration. After a 3-day incubation period, the overlays were aspirated, and the monolayers were fixed using 10% neutral-buffered formalin (Fisher Scientific, Hampton, TN, USA) with 0.2% crystal violet (Ricca, Arlington, TC, USA) prior to enumeration.

### 2.4. Plasma Cytokines

The plasma concentrations of granulocyte-macrophage colony stimulating factor (GM-CSF), interferon gamma (IFN-γ), monocyte chemoattractant protein 1 (MCP-1), and interleukins (IL)-2, -4, -6, -8, -10, and -17 were measured using a Milliplex non-human primate kit (MilliporeSigma, St. Louis, MO, USA) as described previously [13]. The upper and lower limits of quantification (ULOQ and LLOQ) were determined for each analyte from the standards included with the kit.

### 2.5. Hematology

Complete blood counts (Sysmex XS1000i, Lincolnshire, IL, USA) and selected serum chemistries (Piccolo General Chemistry 13 kits, Abaxis, Union City, CA, USA), were performed at the indicated timepoints using blood collected in K3 EDTA and SST tubes (BD, San Jose, CA, USA). Due to a lack of published data, the standard ranges of hematologic parameters and serum chemistries for patas monkeys were defined as the mean +/– two standard deviations of all pre-exposure timepoints. The standard ranges for rhesus monkeys were determined from data kept by veterinary staff on subjects housed in the facility.

### 2.6. Histology, In Situ Hybridization, and Immunohistochemistry

Formalin-fixed paraffin-embedded (FFPE) animal tissue sections (5-µm) were used for immunohistochemical staining using the following antibodies: NKG2A (Abcam, Cambridge, MA, USA); Iba1 (Wako, Richmond, VA, USA); MHC1 [Clone EPR1394Y] (Abcam); CD8 (Abcam); and CD3 [Clone 12] (AbD, Serotec Hercules, CA, USA). Staining was performed on the Bond RX platform (Leica Biosystems, Wetzlar, Germany) according to the manufacturer’s protocol. Briefly, sections were baked, deparaffinized, and rehydrated. Epitope retrieval was performed using Leica Epitope Retrieval Solution 1, pH 6.0, heated to 100 °C for 20 min and quenched with hydrogen peroxide prior to the addition of primary antibody. The Bond Polymer Refine Detection kit (Leica Biosystems) was used for chromogen detection. Image analysis was performed on select tissues from all groups to quantify the degree of positive staining. Images were obtained on a bright-field Leica Aperio AT2 slide scanner (Leica Biosystems) and processed using Aperio Image Scope (v12.3) algorithms. For quantification, images of the entire slide were used to prevent sampling bias. The Positive Pixel Count Algorithm was used to assign pixels to intensity ranges of positive (strong (n_sp_), medium (n_p_), and weak (n_wp_)) and negative (n_n_) pixels. The pixels were categorized, and a positive percentage was calculated per image as a fraction of the number of strong positive (n_sp_) pixels to the total number of pixels:

(1) $${\%}_{positive}=\frac{n_{sp}}{n_{total}}\;\times \;100\;=\;\frac{n_{sp}}{\left(n_{sp}+n_{p}+n_{wp}+n_{n}\right)}\;\times \;100$$

After a percentage of positive pixels per slide was obtained for each subject and marker, values were averaged by group, and the significance was assessed in Excel using the T.TEST function. SHFV RNA RNAscope in situ hybridization (ISH) was performed as previously described [23].

### 2.7. Electron Microscopy

Electron microscopy samples were processed and imaged as previously reported [24].

### 2.8. Whole Blood Viral RNA qPCR

Monkey peripheral blood samples were inactivated in 3 volumes of Trizol LS buffer (Thermo Fisher Scientific, Waltham, MA, USA). RNA was extracted using the Qiagen AllPrep 96 kit as described by manufacturer (Qiagen, Valencia, CA, USA) except that each sample was treated with 27 units of DNAse I (Qiagen). The SHFV RNA copy number was determined by RT–qPCR using primers and probes targeting the SHFV gp15 gene. The AgPath-ID One-Step RT–PCR kit (Thermo Fisher Scientific) was used to perform the assay. The primers and Cal Fluor Orange 560/BHQ1-labeled probe were synthetized by LCG Biosearch Technologies (Petaluma, CA, USA). The RT–qPCR reactions were performed in 20 µL reactions using forward primer (5’-CGACCTCCGAGTTGTTCTACCT-3’), reverse primer (5’-GCCTCCGTTGTCGTAGTACCT-3’), and fluorescent probe (5’-CCCACCTCAGCACACATCAAACAGCT-3’). Synthetic DNA (5’-TTTCGCCGAACCCGGCGACCTCCGAGTTGTTCTACCTGGTCCCACCTCAGCACACATCAAACAGCTGCTGATCAGGTACTACGACAACGGAGGCGGAAATCTTTCATATG-3’; LCG Biosearch Technologies, Novato, CA, USA) was used as a standard. The qPCR reactions were run at 50 °C for 10 min, 95 °C for 10 min, 55 cycles of 95 °C for 15 s, and 60 °C for 45 s on a 7900HT Fast Real-Time PCR System (Thermo Fisher Scientific). The data were analyzed using Applied Biosystems 7900HT Fast Real-Time PCR System Software (Thermo Fisher Scientific).

### 2.9. Flow Cytometry

Whole blood was assessed for the following markers: HLA-DR-FITC (BioLegend, San Diego, CA, USA), CD16-APC (BD), PD-1-APC-Cy7 (BioLegend, San Diego, CA, USA), CD3-AF700 (BD), CD11c-PE (BD), CD28-PE-Cy5 (BioLegend), NKG2a-PE-Cy7 (Beckman Coulter, Brea CA, USA), CD163-PE/Dazzle594 (BioLegend), CD4-BV421 (BD), CD14-BV510 (BioLegend), CD20-BV570 (BioLegend), CD8a-BV605 (BD), CD123-BV650 (BD), PD-L1-BV711 (BioLegend), CD95-BV785 (BioLegend), and Ki-67-PerCP-Cy5.5 (BD). Briefly, 100 μL of whole blood was incubated with 100 μL of the marker panel (excluding Ki-67) and incubated for 20 min. The red blood cells were lysed with 1 mL of BD FACSLyse (BD) for 10 min. The cells were washed and fixed for 30 min using BD Cytofix/Cytoperm (BD) and incubated with Ki-67 antibody for 30 min at 4 °C, followed by a final wash in 1× BD Permwash (BD). Data acquisition and analysis was performed with FlowJo version 10.2 (BD). During the acquisition and analysis, species-specific patterns of cell phenotypes were taken into consideration [25]. After filtering for single cell events and removing granulocytes, immune cell populations were assessed according to the features listed in Table 1.

### 2.10. Statistical Analysis

Statistical analyses were performed using Microsoft Excel with the T.TEST function. The significance was defined as a *p*-value of less than 0.05.

### 2.11. Enzyme-Linked Immunosorbent Assays

The lysates from MA-104 cells infected with SHVF or mock infected (media only) were used as substrates. The Immulon 2 HB microplates (Thermo Fisher Scientific, Walkersville, MD, USA) were coated with cell lysates diluted in PBS and incubated overnight at 4 °C. The plates were then washed five times with wash buffer comprised of PBS/0.2% Tween 20 and blocked for 2 h at room temperature with 5% nonfat milk (LabScientific, Highlands, NJ, USA) dissolved in PBS. The plates were then washed five times with wash buffer, and the analyte serum was diluted at 1:50 in PBS/2.5% milk/0.05% Tween 20 was added in duplicate to the corresponding wells. After a 1-h incubation at room temperature, the plates were washed, and horseradish peroxidase-conjugated anti-monkey IgG (Sigma Aldrich, St. Louis, MO, USA; A2054) was added. The plates were then incubated for 1 h at room temperature before washing with wash buffer and adding TMB Substrate (Thermo Fisher Scientific, Walkersville, MD, USA). Following a 10-min incubation, 100 μL of TMB stop solution (Thermo Fisher Scientific, Walkersville, MD, USA) was added to each well, and the plates were read on a SpectraMax Plus 384 plate reader (Molecular Devices, Sunnyvale, CA, USA) at 450 nm.

## 3. Results

### 3.1. Simian Hemorrhagic Fever Virus (SHFV) Infection Results in Mild Clinical Disease in Patas Monkeys

Twelve monkeys were grouped by species into SHFV-inoculated and PBS-inoculated groups, with three animals per group. Inoculations were 1-mL intramuscular injections in the right quadriceps with either 5000 PFU of SHFV-LVR or PBS. The three SHFV-inoculated patas monkeys developed axillary and inguinal lymphadenopathy starting on day 10 PI that persisted until the conclusion of the experiment at 21 days PI. The three SHFV-inoculated rhesus monkeys developed severe disease. Two subjects (“non-survivors”) met clinical endpoint criteria and were euthanized on days 8 and 11 PI, respectively. The third subject (“survivor”) survived until the conclusion of the experiment (day 20 PI). Signs of disease were first detectable on day 4 PI in SHFV-inoculated rhesus monkeys. They developed a range of clinical signs including gingival bleeding (1/3 subjects), inguinal lymphadenopathy (1/3), and axillary lymphadenopathy (2/3). All the SHFV-inoculated rhesus monkeys developed petechial rashes and axial and inguinal lymphadenopathy by their respective endpoints. All three SHFV-inoculated rhesus monkeys developed tremors and motor dysfunction by day 6 PI that remained until each subject’s respective endpoint. The non-surviving rhesus monkeys developed facial edema that began on day 8 PI that persisted to their respective endpoints. The surviving SHFV-inoculated rhesus monkey developed facial edema that began on day 12 PI and resolved by day 16 PI. All mock-inoculated rhesus monkeys appeared clinically normal and displayed no outward signs of disease throughout the experiment. Animal body weight remained within normal limits for all subjects during the experiment.

### 3.2. SHFV Infection Results in Clinical Pathology Changes in Patas and Rhesus Monkeys

**Figure 1:**
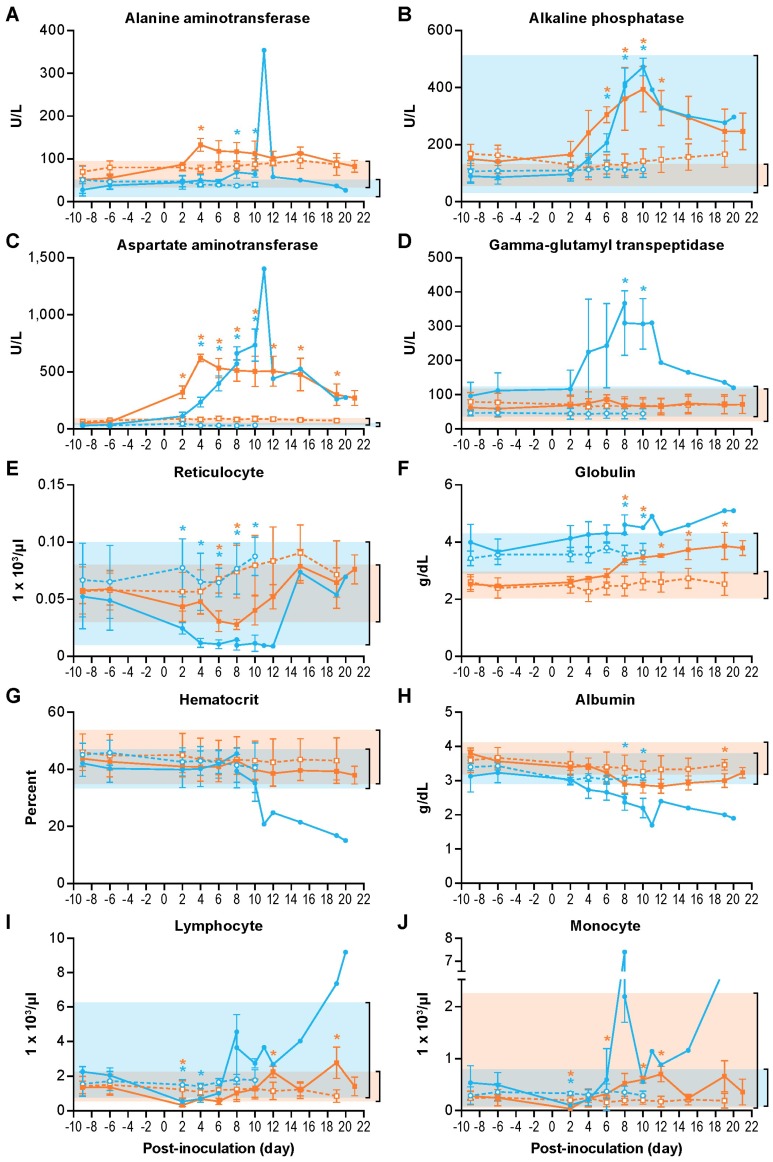
Clinical pathology changes during simian hemorrhagic fever virus (SHFV) infection. Alanine aminotransferase (**A**), alkaline phosphatase (**B**), aspartate aminotransferase (**C**), gamma-glutamyltransferase (**D**), reticulocyte number (**E**), globulin (**F**), hematocrit (HCT) (**G**), albumin (**H**), lymphocyte number (**I**), monocyte number (**J**) values for patas monkeys (orange lines) and rhesus monkeys (blue lines) either inoculated with 5,000 PFU of SHFV (closed symbols) or with PBS (open symbols). The shaded regions represent the standard range of all pre-exposure values for the patas monkeys or previously collected values for the rhesus monkeys. The data represent the means of each group. The error bars represent standard deviation. * symbols represent *p* < 0.05 and are colored according to species.

Indicated serum chemistry analytes were selected to assess organ function and other physiological functions during SHFV infection. In SHFV-inoculated patas monkeys, alanine aminotransferase (ALT, day 4 PI), alkaline phosphatase (ALP, days 6–12 PI) and aspartate aminotransferase (AST, days 6–19 PI) concentrations were significantly elevated on the indicated days (Figure 1A–C). γ-glutamyl transferase (GGT) concentrations remained normal in all SHFV-inoculated patas monkeys (Figure 1D). SHFV-inoculated rhesus monkeys showed similar trends as patas monkeys except that GGT concentrations were elevated on days 8 and 10 PI. ALP, AST, and GGT concentrations remained elevated in the surviving SHFV-inoculated rhesus monkey until the conclusion of the experiment, whereas ALT returned to baseline concentrations. Although changes in serum chemistries were observed in all SHFV-inoculated subjects, the values did not reach concentrations suggestive of a severe clinical disease. All subjects experienced decreases in albumin concentration coinciding with an increase in globulin concentrations (Figure 1F) on days 8–19 and days 8 and 10 PI in SHFV-inoculated patas and rhesus monkeys, respectively. Reticulocyte counts decreased in both SHFV-inoculated patas (days 6 and 8 PI) and rhesus monkeys (days 2–10 PI) (Figure 1E) but did not drop below the normal range. Hematocrit (HCT) remained normal in all subjects except for the surviving SHFV-inoculated rhesus where HCT decreased starting on day 10 PI with anemia persisting to study end (Figure 1G). Albumin concentrations (Figure 1H) were significantly decreased on day 19 PI in SHFV-inoculated patas monkeys. In SHFV-inoculated rhesus monkeys, albumin concentrations were significantly decreased on days 8 and 10 PI. No significant changes in serum chemistry were observed in mock-inoculated patas and rhesus monkeys.

The complete blood counts revealed minor decreases in the lymphocyte numbers early in all SHFV-inoculated patas and rhesus monkeys (days 2 and 2–4 PI, respectively), and were elevated in SHFV-inoculated patas monkeys on days 12 and 19 respectively (Figure 1I). The monocyte counts decreased slightly in all SHFV-inoculated patas and rhesus monkeys (days 2, 6 and day 2 PI, respectively) (Figure 1J). In SHFV-inoculated patas monkeys, the monocyte counts were elevated on days 10 and 12 PI. No significant changes were observed in the complete blood counts in any mock-inoculated subject.

### 3.3. SHFV Infection Results in Clinical Pathology Changes in Patas and Rhesus Monkeys

Gross examination of SHFV-inoculated patas monkeys during necropsy revealed no remarkable findings. Non-surviving SHFV-inoculated rhesus monkeys had marked hepatosplenomegaly: the hepatic tissues were friable and firm. Moderate peripheral and visceral lymphadenopathy were found in both non-surviving rhesus monkeys, whereas moderate peripheral lymphadenopathy was the only significant finding in the surviving rhesus monkey. The kidneys of one non-surviving SHFV-inoculated rhesus monkey contained multiple infarctions with severe renal hemorrhage and necrosis. No significant gross lesions were observed in either mock-inoculated group.

**Figure 2:**
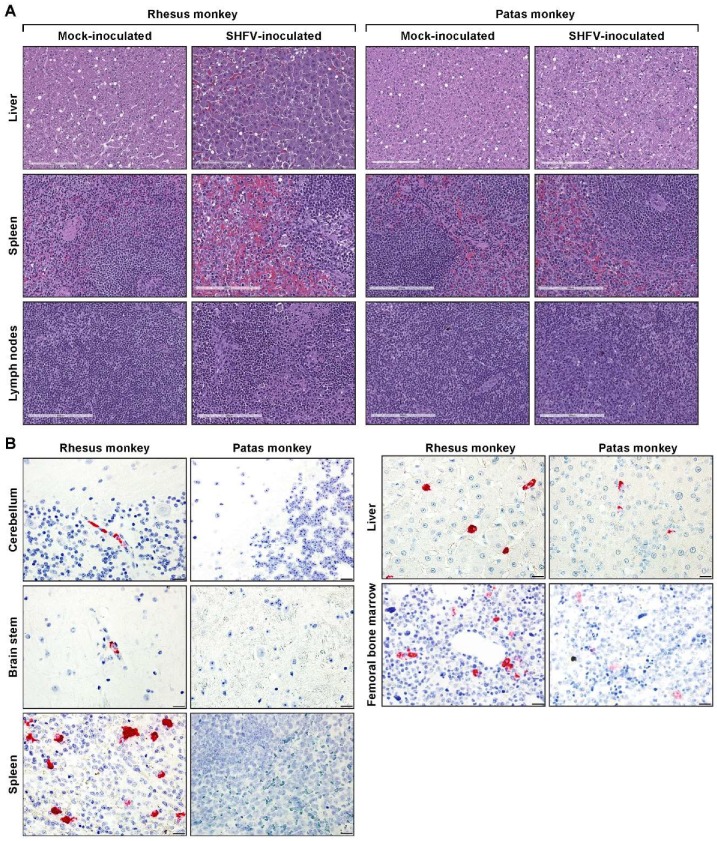
Representative images of hematoxylin- and eosin- (H&E) stained livers, spleens, and lymph nodes of mock- and SHFV-inoculated patas and rhesus monkeys (**A**). Representative images of in situ hybridization used to detect SHFV RNA in terminal cerebellum, brain stem, spleen, femoral bone marrow, and liver samples from patas and rhesus monkeys (**B**). Scale bars represent 200 µm (**A**) and 50 µm (**B**).

The histopathological examination (Figure 2A,B) of the spleen in SHFV-inoculated patas monkeys revealed abundant plasma cells in one subject. The spleens of the two remaining SHFV-inoculated patas monkeys were within normal limits. The livers of two SHFV-inoculated patas monkeys showed inflammatory changes, whereas the third was normal. Hyperplasia was evident in the inguinal lymph nodes of one SHFV-inoculated patas monkey. The spleens of SHFV-inoculated rhesus monkeys were different in each subject. In the two non-survivors, one was congested, whereas fibrin deposition was evident in the other. The spleen of the surviving subject exhibited changes that were consistent with reactive lymphoid hyperplasia, characterized by diffuse expansion and proliferation of B-cells at the margins of each follicle. Each of the livers of SHFV-inoculated rhesus monkeys were also histologically different. In the survivor, perivascular inflammation with multifocal areas of necrosis were evident. The major finding in the liver of one non-survivor was vacuolated hepatocytes, whereas rare thrombi were the major observation in the remaining non-survivor. Hyperplasia was present in the inguinal lymph nodes of all three SHFV-inoculated rhesus monkeys. The tissues of all mock-inoculated subjects were normal apart from vascular congestion in two patas monkey spleens. While all three SHFV-inoculated rhesus monkeys had neurological signs, on histopathological examination, CNS tissues did not reveal any evidence of vasculitis or other changes that would suggest encephalitis. The CNS tissues of all mock-inoculated animals and SHFV-inoculated patas monkeys were found to be within normal histologic limits. Upon histopathological examination, the kidneys of all subjects appeared to be within normal histologic limits.

**Figure 3:**
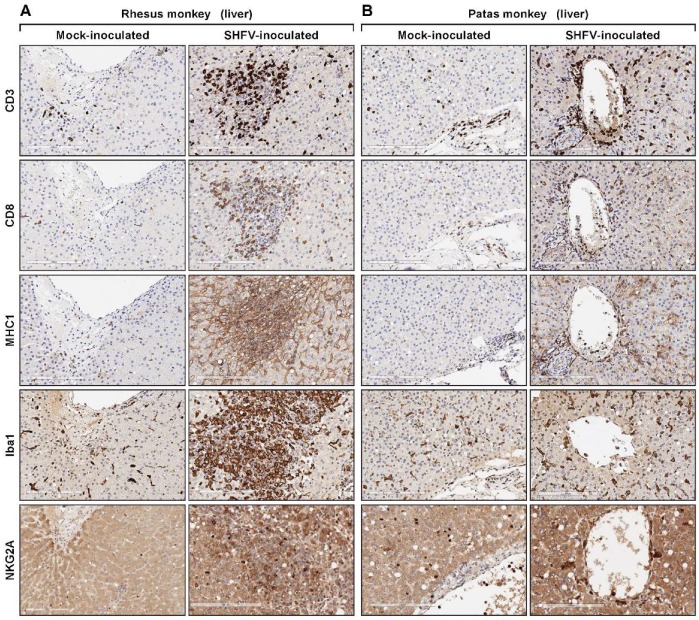
Representative images showing liver immunohistochemistry staining of the indicated markers for SHFV-inoculated rhesus (**A**) and patas (**B**) monkeys Scale bar represents 200 µm.

Immunohistochemical staining (Figure 3) used to detect macrophages expressing ionized calcium-binding adaptor 1 (Iba1) revealed morphologically-normal macrophages in the livers of all three SHFV-inoculated patas monkeys. In contrast, the livers of all three SHFV-inoculated rhesus monkeys contained macrophages that were often rounded and contained a diffusely vacuolated cytoplasm. These changes in macrophage morphology were in direct contrast to those cells in infected patas monkeys, which often exhibited a more stellate shape and cytoplasm that was diffusely dark brown when evaluated immunohistochemically. Similar findings were seen in the inguinal lymph nodes and spleen of SHFV-inoculated patas and rhesus monkeys, although rounded macrophages were detected in the spleen of one SHFV-inoculated patas monkey and inguinal lymph nodes of a second SHFV-inoculated patas monkey. The macrophages appeared morphologically normal in all mock-inoculated subjects.

### 3.4. Viral RNA (vRNA) Replication Is Sustained in SHFV-Infected Patas Monkeys

**Figure 4:**
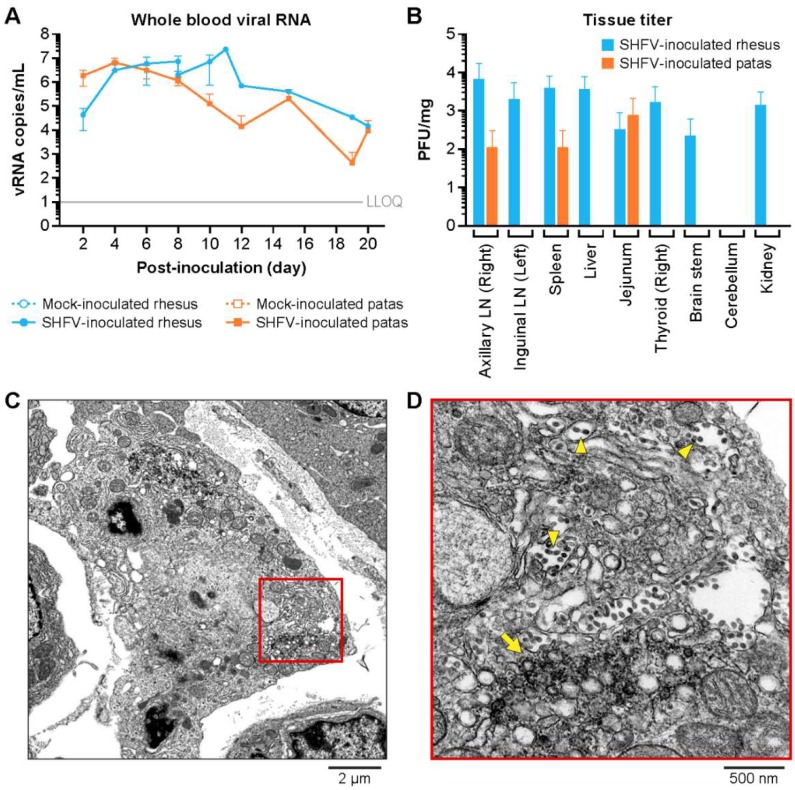
Virological and microscopic evidence of SHFV replication in patas and rhesus monkeys. Mean vRNA copy number values in viral RNA copies per mL of whole blood for mock (open symbols) and SHFV-inoculated (closed symbols) patas monkeys (orange) and rhesus monkeys (blue) (**A**). The mean titer of tissues for SHFV-inoculated patas monkeys (orange) and rhesus monkeys (blue) in PFU per mg of 10% tissue homogenate of lymph nodes (LN), spleen, jejunum, cerebellum, and kidneys (**B**). Electron micrograph of jejunum from a SHFV-inoculated patas monkey showing double-membrane vesicles (DMVs) and apparently mature virions (red box, C). Enlargement of boxed area from (**C**) showing apparently mature virions (yellow arrowheads) and double-membrane vesicles (yellow arrow) (**D**). The error bars represent standard deviation.

SHFV-inoculated patas and rhesus monkeys had detectable viral RNA (vRNA) in the circulating blood on day 2 PI (Figure 4A). The average peak titers in patas and rhesus monkeys were 6.75 (range 6.41–6.96) and 7.08 (range 6.79–7.36) log_10_ vRNA copies per mL, respectively. The patas monkeys reached the peak vRNA copy number on day 4 PI, whereas the rhesus monkeys reached the peak vRNA copy number between days 5 and 12 PI. In patas monkeys, vRNA copies were detectable in all three subjects for the remainder of the experiment, except for days 15 and 19 PI when vRNA copy number was below the limit of detection in two subjects. The terminal vRNA copy number in SHFV-inoculated patas monkeys was 3.42 (range 2.51–4.43) log_10_ vRNA copies per mL whole blood. The terminal vRNA copy numbers in SHFV-inoculated rhesus monkeys was 6.30, 7.36, and 4.17 log_10_ vRNA copies per mL in the two non-survivors and single survivor, respectively. The correlation of vRNA copies per mL with survival or clinical parameters is unwarranted due to the survival of 1 of the SHFV-inoculated rhesus monkeys.

SHFV was detected by plaque assay in the axillary lymph node (*n* = 1), spleen (*n* = 1), and jejunum (*n* = 1) of two SHFV-inoculated patas monkeys (Figure 4B). The highest titer was 3.36 log_10_ PFU/mg in the jejunum of the SHFV-inoculated patas with the highest terminal vRNA copy number. In the two non-surviving SHFV-inoculated rhesus monkeys, SHFV was found in the axillary and inguinal lymph nodes (*n* = 2 and 1, respectively), spleen (*n* = 2), liver (*n* = 2), jejunum (*n* = 1), thyroid (*n* = 2), brain-stem (*n* = 1), and kidneys (*n* = 2). Kidney tissue was the only tissue positive in all three SHFV-inoculated rhesus monkeys. The highest tissue titer in SHFV-inoculated rhesus monkeys was 4.27 log_10_ PFU/mg in the axillary lymph nodes. Plaque assays on whole blood were not performed due to experimental sample requirements and blood collection volume limits.

The bone marrow, cerebella, jejuna, axial and inguinal lymph nodes, kidneys, and thyroids were assessed for evidence of SHFV infection by transmission electron microscopy (TEM). Double membrane vesicles (DMVs) and apparently mature virions were found in the jejunum of the SHFV-inoculated patas monkey with the highest terminal vRNA copy number (Figure 4C,D). The livers of SHFV-inoculated patas monkeys were negative and were therefore not examined by TEM. ISH for SHFV vRNA was performed to assess the cerebella, brainstems, spleens, livers and femoral bone marrow of SHFV-inoculated subjects for signs of SHFV replication. The livers of two SHFV-inoculated patas monkeys and, rarely, the spleen of the third were positive for SHFV vRNA (Figure 2B) using RNAscope. The femoral bone marrow of the patas monkey with the highest terminal titer was positive for vRNA. vRNA was detected by ISH in the cerebellum, brain-stem, spleen, and liver of all SHFV-inoculated rhesus monkeys. vRNA was detected in femoral bone marrow of all three SHFV-inoculated rhesus monkeys. Morphologically, ISH data support that monocytes and endothelial cells are sites of SHFV infection in examined livers, spleens, brainstems, and cerebella of both SHFV-inoculated patas and rhesus monkeys. In all tissues, cells positive for SHFV vRNA (RNAScope) were morphologically consistent with macrophage-lineage cells. In each of the tissues evaluated, these cells were present in fairly low numbers.

### 3.5. SHFV Infection of Patas and Rhesus Monkeys Elicits Strong and Overlapping Immune Responses

**Figure 5:**
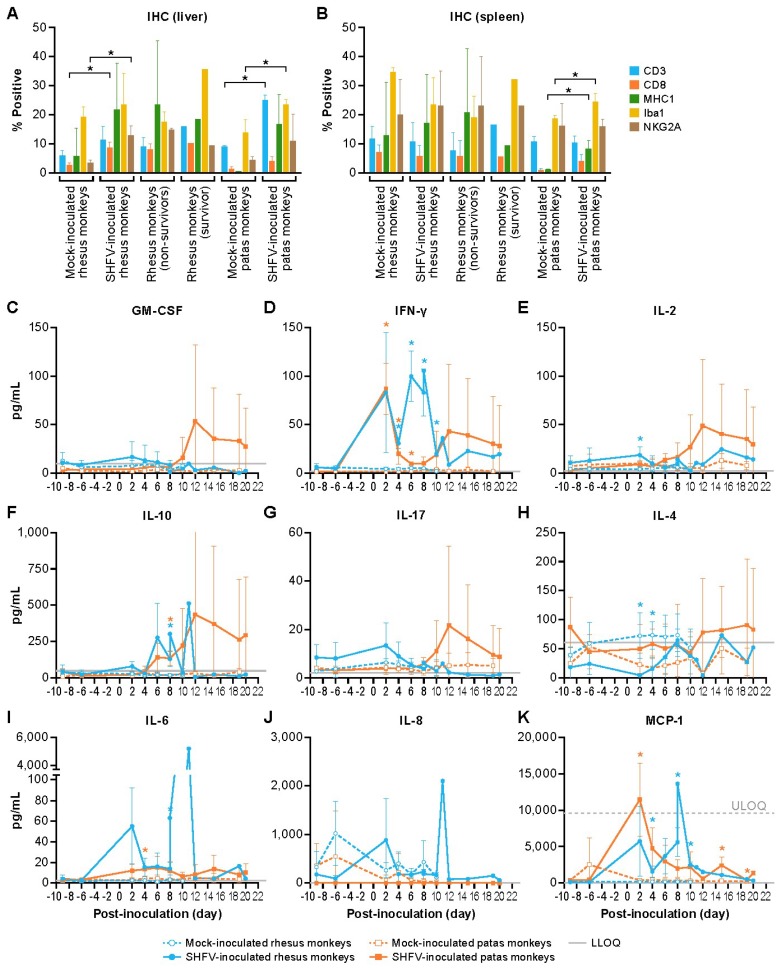
Immunophenotype of the liver and spleen (**A**,**B**) and plasma cytokine concentrations of SHFV-infected and mock-infected animals. The mean quantitative immunohistochemistry values of the indicated marker in mock- and SHFV-inoculated patas and rhesus monkey livers and spleens (**A**,**B**). The mean plasma concentrations in pg per mL of indicated analyte for mock (open symbols) and SHFV-inoculated (closed symbols) patas monkeys (orange) and rhesus monkeys (blue) (**C**–**K**). The gray lines represent the lower and upper limits of quantitation (LLOQ and ULOQ, respectively). The error bars represent standard deviation. * symbols represent *p* < 0.05 and are colored according to species.

Quantitative immunohistochemistry (qIHC) (Figure 5) revealed statistically significant (*t*-test, *p* < 0.05) changes in inflammatory cell populations in the livers (Figure 3) of SHFV-inoculated monkeys (Figure 5A). On average, SHFV-inoculated patas monkeys had livers with increased CD3 and Iba1 signals when compared to uninfected patas monkeys. CD8 signals were increased in the liver of SHFV-inoculated patas monkeys when compared to uninfected patas monkeys, but the difference did not reach significance (*p* = 0.06). CD8 and natural killer group protein 2a (NKG2A), a natural killer (NK) cell marker, signals were increased in SHFV-inoculated rhesus monkeys compared to uninfected controls. In the spleen, significant changes were seen in major histocompatibility complex class-1 (MHC-1) and Iba1 signals between SHFV-inoculated and mock-inoculated patas monkeys (Figure 5B). No significant differences in cell frequencies were observed for CD3, CD8, and NKG2A in the splenic tissue of infected and uninfected patas monkeys. No significant changes were seen between SHFV-inoculated and mock-inoculated rhesus monkey spleens for any of the markers quantified.

Analysis of plasma cytokine concentrations (Table 2) identified changes in both patas and rhesus monkeys during SHFV infection. Interferon gamma (IFN-γ) concentrations in all SHFV-inoculated patas monkeys were elevated on day 2 PI compared to the pre-exposure mean concentration (group mean fold change (Figure 5D)). Later, IFN-γ concentrations decreased to baseline in two SHFV-inoculated patas monkeys, whereas the concentration of the third patas monkey remained elevated throughout the experiment with a second peak in concentration at 12 days PI. The two non-surviving SHFV-inoculated rhesus monkeys had peak concentrations of similar magnitudes 2 days PI, and all three subjects had increased concentrations by day 6 PI. The concentration of IL-2 (Figure 5E) was significantly increased on day 2 PI in SHFV-inoculated rhesus monkeys. IL-10 concentrations (Figure 5F) were significantly increased in the animals of both species on day 10 PI. IL-4 concentrations (Figure 5H) were significantly decreased on days 2 and 4 PI. However, all SHFV-infected rhesus monkey cytokine concentrations for these days were below the lower limit of detection. The SHFV-inoculated patas monkeys had mild increases in interleukin 6 (IL-6) concentrations in comparison to their mock counterparts but only significantly so on day 4 PI. SHFV-inoculated rhesus monkeys had increased IL-6 concentrations starting on day 2 PI, but these concentrations only reached statistical significance on day 8 PI (survivor) and terminal days (two non-survivors) (Figure 5I). Mean monocyte chemoattractant protein 1 (MCP-1, Figure 5K) concentrations peaked at day 2 PI in all SHFV-inoculated patas monkeys and the two non-surviving rhesus monkeys. All SHFV-inoculated rhesus monkeys had a second MCP-1 concentration peak at day 8 PI. GM-CSF, IL-17, and IL-8 concentrations were not significantly increased at any of the analyzed time points (Figure 5A,G,J, respectively).

**Figure 6:**
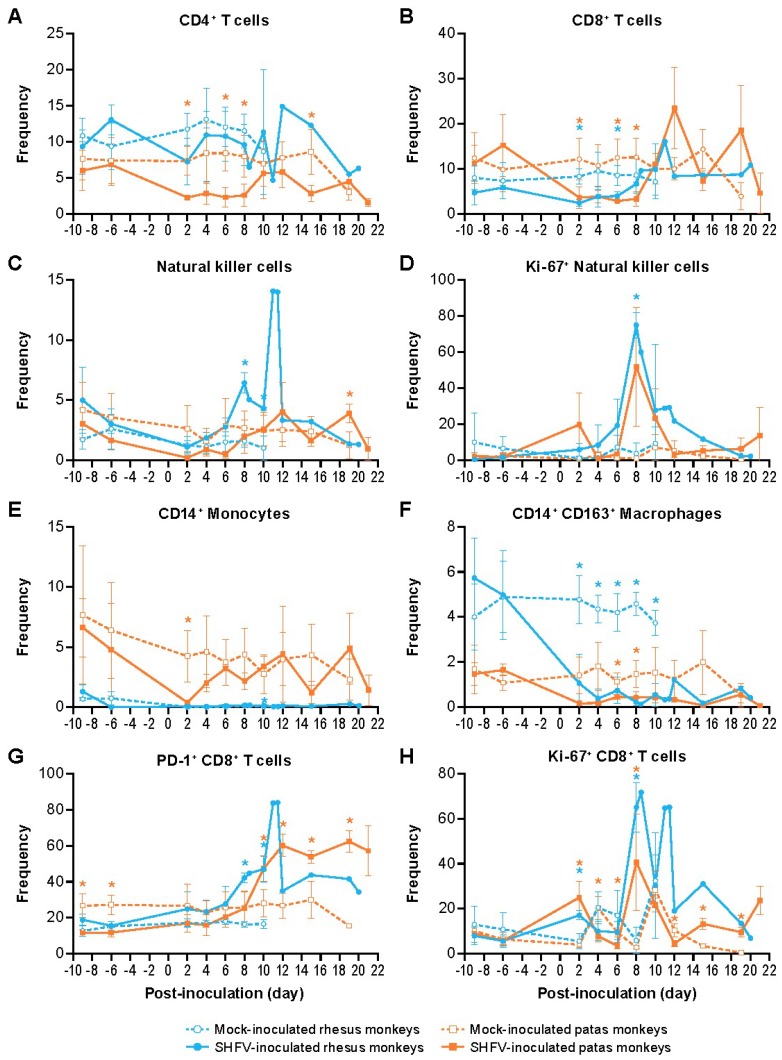
Circulating cell populations in SHFV-infected and mock-infected animals. The mean frequency of the indicated cell populations from whole blood of mock- (closed symbols) and SHFV-inoculated (open symbols) patas monkeys (orange) and rhesus monkeys (blue) (**A**–**H**). The error bars represent standard deviation. * symbols represent *p* < 0.05 and are colored according to species.

Flow cytometry of whole blood revealed that CD4^+^ T-cells were decreased in SHFV-inoculated patas monkeys on days 2–8 and day 15 PI. CD4^+^ T-cell numbers did not significantly changed in SHFV-inoculated rhesus monkeys (Figure 6A). CD8^+^ T-cell numbers were decreased in both SHFV-inoculated patas and rhesus monkeys (days 2–8 and days 2, 6 PI, respectively) (Figure 6B). Increased frequency of circulating NK cells were observed in SHFV-inoculated patas monkeys on day 19 PI (Figure 6C). In contrast, SHFV-inoculated rhesus monkeys had a single, larger, increase in circulating NK cells on days 8 to 10 PI. The changes in Ki67^+^ NK cells in SHFV-inoculated patas monkeys were more variable, with one patas monkey reaching peak frequency at day 2 PI and the other two patas monkeys reaching peak frequency at day 8 PI (Figure 6D). Ki-67^+^ NK cells were significantly increased only in SHFV-infected rhesus monkeys on day 8 PI.

Circulating CD14^+^ monocytes (Figure 6E) were decreased in SHFV-inoculated patas monkeys at day 2 PI prior to returning to baseline frequency, whereas frequency of these monocytes in SHFV-inoculated rhesus monkeys appeared unchanged throughout the experiment, excluding a small, but significant, increase on day 10 PI (Figure 6E). SHFV-inoculated patas and rhesus monkeys had decreased frequency of CD14^+^ CD163^+^ macrophages (Figure 6F) on days 6–8 PI and 2–10 PI, respectively, and these numbers remained unchanged until each subject’s respective endpoint. The numbers of PD-1^+^ CD8^+^ T-cells began to increase in both SHFV-inoculated patas and rhesus monkeys on days 10–19 and 8–10 PI, respectively (Figure 6G). Ki-67^+^ CD8^+^ T-cell frequency was elevated in SHFV-inoculated patas monkeys from days 2–19 PI with the exception of day 10 PI (Figure 6H). In SHFV-inoculated rhesus monkeys, Ki-67+ CD8+ T-cells were elevated on days 2 and 8 PI.

**Figure 7:**
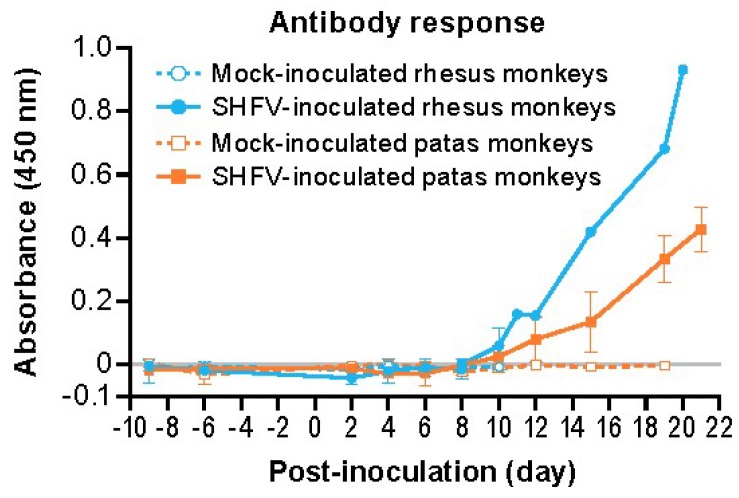
Mean ELISA absorbance values for patas monkeys (orange) and rhesus monkeys (blue) either inoculated with 5000 PFU of SHFV (open symbols) or with PBS (closed symbols). The error bars represent standard deviation.

IgG antibody responses were detected by enzyme-linked immunosorbent assay (ELISA) in all three SHFV-inoculated patas monkeys and two of the three (the survivor and one non-survivor) SHFV-inoculated rhesus monkeys (Figure 7). Two SHFV-inoculated patas monkeys had detectable anti-SHFV antibodies on day 10 PI and the third monkey on day 15 PI. Two of three SHFV-inoculated rhesus monkeys mounted antibody responses by day 10 PI. The response magnitude continued to increase in all responding subjects until their respective endpoints. Anti-SHFV antibodies were not detected in mock-inoculated subjects at any time.

## 4. Discussion

The goal of this experiment was to characterize and compare SHFV infection of patas and rhesus monkeys to assess the usefulness of comparing biologically similar refractory and susceptible primate species in hemorrhagic fever virus infection. This is the first report of successful experimental SHFV infection of patas monkeys. Our data demonstrate that SHFV can replicate to high titers in patas monkeys without causing significant disease. The disease observed in SHFV-inoculated patas monkeys is a mild clinical disease characterized by changes in serum chemistry and circulating blood populations, with transient lymphadenopathy being the only outward sign of infection.

Consistent with previously obtained data derived from experimentally SHFV-infected macaques [10,13,14], ISH and electron microscopy of the infected tissues indicate that tissue-resident macrophages and endothelial cells are likely the main targets of SHFV in patas monkeys. Together, these data suggest that SHFV targets the same cell types in cercopithecines. Although the vRNA copy number peaked earlier in patas monkeys, the peak viral loads were largely similar between all monkeys. In rhesus monkeys and Japanese macaques, the day of peak SHFV titer is highly variable and does not correlate with survival [13,14]. These results suggest that the timing and magnitude of viral load is unlikely to have significant impacts on disease course, although additional studies would be required to fully characterize the relationship between disease phenotype and viral replication dynamics in monkeys of each species.

Given the extreme differences in disease course and outcomes during SHFV infection in patas and rhesus monkeys, the relatively high degree of overlap in host-response features was unexpected. Changes in IFN-γ and MCP-1 concentrations, and circulating macrophage, NK, and T-cell populations, were similar between SHFV-inoculated patas and rhesus monkeys. However, patas monkeys did not respond with large IL-6 concentration increases observed in both this experiment’s rhesus monkeys and in other experimental work [10,13,14]. This difference is of particular interest as IL-6 has been associated with non-survival in SHFV-infected rhesus monkeys, and because decreased concentrations of IL-6 were seen in in vitro infection of monocyte-derived macrophages and dendritic cells from baboons [13,15]. Given the potential role of IL-6 in human VHFs, our model offers an opportunity to explore the potential of therapies, such as neutralizing antibodies, aimed at modulating IL-6 responses during infection [26,27].

Although animals of both species showed decreases in CD8^+^ T-cell frequency early in infection, with an increase in PD1^+^ CD8^+^ T-cell frequency later in infection, only SHFV-infected patas monkeys showed an increase in CD4^+^ T-cell numbers largely throughout infection. Additional cell types or circulating factors not covered in this experiment may be present in monkeys of either species during SHFV infection. Future experiments are required to further define the function and activation state of T and NK cells in monkeys of both species. Functional differences among cell types and circulating factors between patas and rhesus monkeys may also play a role and could be the subject of further experiments.

Although similarities in circulating cell populations were observed in monkeys of both species, significant differences in tissue immune cells were observed. The livers of SHFV-inoculated patas monkeys had increased CD3 and Iba1 IHC signals, whereas those of SHFV-inoculated rhesus monkeys had elevated CD8 and NKG2A IHC signals. Increases in CD8 and NKG2A signals in the absence of an increase in the CD3 signal suggests that SHFV infection leads to an increase in infiltrating NK cells in rhesus monkeys [28]. Indeed, the frequency of circulating NK cells were elevated at days 8 and 10 PI in rhesus monkeys. The data suggest that a potent type 1 IFN response occurs in SHFV-inoculated rhesus monkeys, but whether a similar response is present in SHFV-inoculated patas monkeys is unclear. NK cell responses are important for survival in human Ebola virus disease cases [29,30]. Differences in the timing of NK cell responses are also a key difference in non-lethal, mild disease and lethal, severe Lassa virus infections in macaques [31], suggesting the species-specific NK cell responses observed here warrant further exploration. For comparison, and as described in the recent paper by Buechler et al. [11], olive baboons and rhesus monkeys infected with the SHFV-related Southwest baboon virus 1 (SWBV-1) also developed increases in NK and CD8^+^ T-cell numbers, with CD8^+^ T-cell frequency remained elevated throughout the observation period. Infected olive baboons and surviving rhesus monkeys had less prominent NK cell increases compared to the non-surviving rhesus monkeys. NK cell dynamics suggest a short-lived peak in olive baboons and rhesus monkeys that did not develop severe disease due to SWBV-1 infection. Together, these data support a role for appropriate timing and activation of NK cells in modulating disease presentation in VHFs. While standard ELISA assays are unable to distinguish between neutralizing and non-neutralizing antibodies, recent work has demonstrated that non-neutralizing antibodies are required for protection against Lassa virus and can be used as part of antibody cocktails in treating Ebola virus infections [32,33]. Additional work is required to further characterize the role and importance of anti-SHFV antibodies in both patas and rhesus monkeys.

Increased detection of Iba1, a macrophage marker, suggests that SHFV infection leads to an increase in the number of macrophages in the liver [34]. Determining the sources and function of these additional liver macrophages in SHFV-inoculated patas monkeys is important given the changes in MCP-1 and IL-6 concentrations and the differential roles of hepatic resident and non-resident macrophages [35,36,37,38]. SHVF is dependent on CD163 for cellular entry [39], and preferential targeting of CD163^+^ cells by SHFV may explain why both patas and rhesus monkeys lost CD163^+^ macrophages. CD163 is associated with alternatively-polarized macrophages, and macrophage polarization plays a significant role in immunity and infection [40,41,42]. The presence of rounded and vacuolated macrophages in the livers of SHFV-inoculated rhesus monkeys but not in their patas counter parts suggests that a species-specific macrophage function may play a key role in SHFV pathogenesis.

Rather than a “cytokine storm”, we propose that a process driven by NK cells and macrophages is the deciding factor in developing hemorrhagic fever. The lack of massive differences in host response to SHFV infection in each species suggests that a relatively small subset of the host response has the potential to avoid or cause hemorrhagic fever during viral infection. Similar results have been observed when comparing hemorrhagic and non-hemorrhagic variants of lymphocytic choriomeningitis virus (LCMV) in rhesus monkeys. Additionally, hemorrhagic LCMV infections in rhesus monkeys share features with SHFV infections in rhesus monkeys, such as increased IL-6 concentrations and an early peak in NK cells [43,44,45]. Both variants of LCMV induced strong host responses, but only a small number of differentially expressed genes were identified between hemorrhagic and non-hemorrhagic infections [45,46]. This work with LCMV and our work suggest that viral hemorrhagic fevers may be initiated by relatively small components of the host’s response to infection. As such, the comparison of SHFV infection in patas and rhesus monkeys offers a useful tool to characterize mechanisms involved in developing hemorrhagic fevers and exploring or developing therapies to treat human disease. SHFV infection in patas and rhesus monkeys could be used as a tool to explore the use of neutralizing antibodies that target immune system components, both to develop therapies and further characterize mechanisms of disease.

## Figures and Tables

**Table 1:** Flow cytometry gating strategy.

Leukocyte Subset	Phenotype
T Cells	NOT Granulocytes, CD3^+^
CD4^+^ T Cells	CD3^+^, CD4^+^
CD4^+^ Naive	CD3^+^, CD4^+^, CD28^+^, CD95^−^
CD4^+^ Central Memory	CD3^+^, CD4^+^, CD28^+^, CD95^+^
CD4^+^ Effector Memory	CD3^+^, CD4^+^, CD28^−^, CD95^+^
CD8^+^ Cells	CD3^+^, CD8^+^
CD8^+^ Naive	CD3^+^, CD8^+^, CD28^+^, CD95^−^
CD8 Central Memory	CD3^+^, CD8^+^, CD28^+^, CD95^+^
CD8 Effector Memory	CD3^+^, CD8^+^, CD28^−^, CD95^+^
B Cells	CD20^+^
CD14^+^ Monocytes	HLA-DR^+^, CD14^+^, CD163^−^
CD14^+^CD163^+^ Macrophages	HLA-DR^+^, CD14^+^, CD163^+^
CD14^+^CD163^+^ Macrophages	HLA-DR^+^, CD14^−^, CD163^+^
Myeloid Dendritic Cells (mDCs)	HLA-DR^+^, CD14^−^, CD163^−^, CD11c^+^, CD123^−^
Plasmacytoid Dendritic Cells (pDCs)	HLA-DR^+^, CD14^−^, CD163^−^, CD11c^−^, CD123^+^
Natural Killer Cells (NK)	HLA-DR^−^, CD3^−^, CD20^−^, SSClow, CD8^+^

**Table 2:** Serum cytokine concentrations.

Analyte	Group	Mean Day of Peak (Range)	Mean Peak Concentration (Range) (pg/mL)	Mean Fold Change from Pre-Exposure Mean (Range) (pg/mL)	No. NHPs with Peak Concentration at Endpoint
GM-CSF	Rhesus-Mock	−1 (−9–4)	12.88 (11.47–14.18)	0.73 (0.1–1.4)	0/3
	Patas-Mock	3 (−9–10)	7.26 (4.21–11.62)	0.83 (0.1–2.39)	0/3
	Rhesus-SHFV	3.67 (-6–15)	17.69 (5.71–34.58)	0.97 (0.05–2.15)	0/3
	Patas-SHFV	14.34 (12–19)	53.96 (7.08–144.34)	12.37 (0.01–94.85)	0/3
IFNγ	Rhesus-Mock	−0.34 (−9–6)	7.2 (5.51–8.99)	0.45 (0.05–0.89)	0/3
	Patas-Mock	10.34 (6–15)	5.04 (3.87–6.3)	0.82 (0.12–2.8)	0/3
	Rhesus-SHFV	5.34 (2–8)	121.72 (97.01–145.68)	8.45 (1.15–29.66)	0/3
	Patas-SHFV	5.34 (2–12)	89 (70.41–122.97)	17.34 (0.45–80.81)	0/3
IL-2	Rhesus-Mock	6 (4–8)	11.03 (9.1–12.21)	0.52 (0.25–1.47)	0/3
	Patas-Mock	3.67 (−6–15)	15.16 (7.79–21.71)	2.78 (0.77–10.43)	0/3
	Rhesus-SHFV	3.67 (−6–15)	25.48 (24.5–27.41)	1.96 (0.22–7.49)	0/3
	Patas-SHFV	15.34 (12–19)	51.96 (9.27–127.81)	14.64 (0.45–83.99)	0/3
IL-10	Rhesus-Mock	−4.34 (−9–2)	49.02 (41.91–57.38)	2.35 (0.93–4.12)	0/3
	Patas-Mock	11 (4–19)	57.66 (35.36–86.99)	10.42 (0.19–49.3)	1/3
	Rhesus-SHFV	8.34 (6–11)	394.88 (149.65–522.9)	14.36 (2.01–57.89)	1/3
	Patas-SHFV	9.34 (6–12)	515.1 (118.66–1240.73)	125.39 (1.1–815.32)	0/3
IL-17	Rhesus-Mock	5.34 (2–10)	7.64 (7.09–7.91)	0.55 (0.2–0.86)	1/3
	Patas-Mock	15.34 (12–19)	6.37 (3.44–11.18)	1.25 (0.02–2.56)	1/3
	Rhesus-SHFV	4 (2–8)	13.47 (3.69–22.07)	0.71 (0.21–1.6)	0/3
	Patas-SHFV	9.34 (6–12)	23.04 (4.51–59.42)	5.31 (0.32–39.05)	0/3
IL-4	Rhesus-Mock	5.34 (2–8)	107.71 (100.81–117.81)	7.5 (3.13–14.18)	0/3
	Patas-Mock	14.67 (10–19)	65 (51.98–75.98)	9.61 (0.75–29.45)	1/3
	Rhesus-SHFV	8 (6–10)	85.2 (71.17–97.69)	6.4 (0.3–29.86)	0/3
	Patas-SHFV	6 (−9–19)	167.85 (136.68–220.38)	33.54 (0.12–144.82)	0/3
IL-6	Rhesus-Mock	4.67 (2–8)	3.96 (3.65–4.5)	0.27 (0.03–0.45)	0/3
	Patas-Mock	4.67 (−9–15)	6.25 (3.14–10.5)	1.07 (0.43–2.38)	0/3
	Rhesus-SHFV	9 (8–11)	1765.16 (20.34–5212.14)	19.84 (0.79–322.88)	1/3
	Patas-SHFV	7.67 (2–15)	22.41 (18.46–28.86)	5.27 (0.21–18.96)	0/3
IL-8	Rhesus-Mock	−3.34 (−6–2)	1062.94 (440.5–1640.1)	30.11 (5.28–112.68)	0/3
	Patas-Mock	−3.67 (−9–4)	556.13 (1.53–1640.1)	7.95 (0–103.88)	0/3
	Rhesus-SHFV	5 (2–11)	1520.19 (610.34–2102)	59.82 (10.78–564.94)	1/3
	Patas-SHFV	15 (12–21)	4.53 (2.3–8.99)	0.92 (0.01–5.91)	1/3
MCP-1	Rhesus-Mock	2 (−6–8)	237.47 (204.28–294.46)	21 (13.69–27.62)	0/3
	Patas-Mock	2 (−6–8)	2681.4 (459.13–6776.22)	132.74 (34.86–458.34)	0/3
	Rhesus-SHFV	6 (2–8)	10549.99 (7716.58–13654.57)	503.84 (102.16–2358.69)	0/3
	Patas-SHFV	2 (2–2)	11477.69 (5959.96–15828.29)	1074.52 (85.91–5030.32)	0/3
